# Pathogenicity and virulence of *Clostridioides difficile*

**DOI:** 10.1080/21505594.2022.2150452

**Published:** 2023-01-04

**Authors:** Jessica E. Buddle, Robert P. Fagan

**Affiliations:** Molecular Microbiology, School of Biosciences, University of Sheffield, Sheffield, UK

**Keywords:** *c. difficile*, virulence factors, antimicrobial resistance, toxins, spores, CDI

## Abstract

*Clostridioides difficile* is the most common cause of nosocomial antibiotic-associated diarrhea, and is responsible for a spectrum of diseases characterized by high levels of recurrence, morbidity, and mortality. Treatment is complex, since antibiotics constitute both the main treatment and the major risk factor for infection. Worryingly, resistance to multiple antibiotics is becoming increasingly widespread, leading to the classification of this pathogen as an urgent threat to global health. As a consummate opportunist, *C. difficile* is well equipped for promoting disease, owing to its arsenal of virulence factors: transmission of this anaerobe is highly efficient due to the formation of robust endospores, and an array of adhesins promote gut colonization. *C. difficile* produces multiple toxins acting upon gut epithelia, resulting in manifestations typical of diarrheal disease, and severe inflammation in a subset of patients. This review focuses on such virulence factors, as well as the importance of antimicrobial resistance and genome plasticity in enabling pathogenesis and persistence of this important pathogen.

## Introduction

*Clostridioides difficile* is a gram-positive obligate anaerobe, capable of causing disease through the fecal-oral transmission of robust endospores. These metabolically dormant spores are able to persist in a range of environments, being resistant to oxygen, heat, and many common disinfectants – contributing to both the organism’s success as a pathogen, and the associated healthcare costs and difficulty of treating infection [1]. *C. difficile* is responsible for over 120,000 infections per year in the EU alone [2] and is the leading cause of hospital-associated diarrhea. As well as being an important nosocomial pathogen, a recent paradigm-shift has seen increasing reports of community-acquired *C. difficile* infection (CDI). Although often less severe, community-acquired CDI is responsible for an estimated 20–27% of all cases, resulting in a significant burden [3]. Clinical presentation of CDI covers a large spectrum of diseases, with diarrhea and colitis being the most common. The significant mortality associated with *C. difficile* typically arises from more severe manifestations, including pseudomembranous colitis, fulminant colitis, and toxic megacolon [4]. Infection recurrence, characterized by the reappearance of symptoms after treatment completion, is also common, largely due to the nature of available CDI treatments. This results in complex treatment plans and worsened prognosis [5,6].

Paradoxically, antibiotics constitute both the main treatment and a main risk factor for *C. difficile* infection. Administration of broad-spectrum antimicrobials, either prophylactically or to treat another infection, lead to disruption of the gut microbiota, resulting in a dysbiotic state in which *C. difficile* thrives [7]. As well as being associated with the broad-spectrum antimicrobials cephalosporins, clindamycin, and fluoroquinolones; antibiotics commonly used to treat *C. difficile* itself can also contribute to CDI, by exacerbating dysbiosis and leaving the patient acutely sensitive to reinfection or relapse [8]. This recurrence is the most common complication of CDI, arising in up to 30% of patients [9]. Collectively, this combination of factors warrants the recent classification of *C. difficile* as an “urgent threat” [10]. This review provides an overview of the *C. difficile* lifecycle, virulence factors, and antimicrobial resistance in the context of pathogenicity.

## The changing epidemiology of *C. difficile*

The phylogenetic diversity of *C. difficile* has allowed for the emergence of several epidemic strains in recent years. In particular, the ribotype 027 lineage was responsible for a 2001 North American epidemic, which spread to the UK, peaking in 2004–2006 [11]. This hypervirulent lineage is associated with increased transmission and mortality, although the underlying reasons for the apparent increase in pathogenicity are far from clear. Ribotype 027 strains display increased expression of toxins, due to a deletion in *tcdC* (encoding a negative regulator of toxin expression) [12–14], and production of an additional binary toxin [15]. These strains also displayed high-level resistance to fluoroquinolone antibiotics, including the commonly used ciprofloxacin [16]. In the UK, improved hospital management strategies have resulted in an 80% reduction in cases from the height of the 2004–2006 outbreak, with current annual infection rates lying at 13,000 [17; 18]. *C. difficile* is responsible for an estimated 29,000 and 1,800 deaths per year in the USA and UK, respectively, [19; 20], with a case fatality rate of approximately 15%. However, this increases with each subsequent infection recurrence [5]. Despite strategies to reduce CDI, the costs associated with CDI have increased, with infections costing between $436 million to $3 billion per year in the USA, with total CDI-attributable costs excelling $6.3 billion [21]. In England, CDI costs an additional £5,000-£15,000 per case [21]. This burden is not solely economic – with the average UK patient stay being 37 days, CDI puts huge pressures on healthcare facilities [22]. Despite less emphasis being placed on the burden of CDI in lower-income countries, it is clear that the lack of diagnosis and prevention has led to a severe underestimation of CDI. In many African countries, due to reduced regulation of antibiotics and high HIV prevalence, CDI burden is likely to be high [23]. Similarly, in South Africa, CDI incidence was shown to be 9.2%, a third of which was community-acquired [24].

## Life cycle and disease transmission

**Figure 1.**
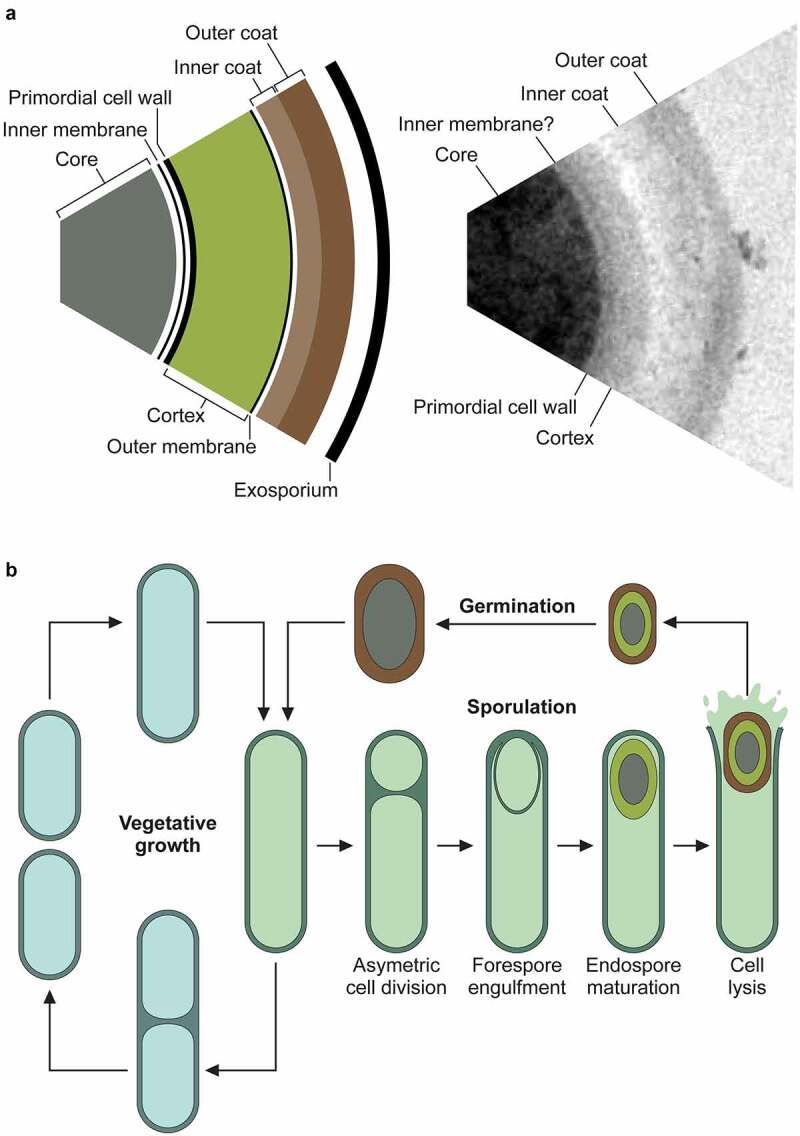
Structure of the spore and life cycle.

As an anaerobe, *C. difficile* must overcome the formidable barrier of atmospheric oxygen to spread to a new host. This is achieved through the formation of metabolically inert but incredibly resilient spores (Figure 1(a)). Mutants defective in sporulation do not transmit efficiently in an animal model that closely mimics both direct patient to patient spread and infection from contaminated surfaces in the environment [28]. In addition to providing resistance to oxygen, the spore form is also resistant to UV, desiccation, heat, many disinfectants, and antibiotics, which creates significant additional decontamination challenges in health-care facilities and also dramatically extends the maximum time interval between hosts [29; 30]. This complicates outbreak control and analysis of chains of transmission by traditional methods [31]. The efficiency of sporulation also varies widely between *C. difficile* strains [32] and it has been suggested that this feeds into the differences observed in transmission. Early in the ribotype 027 epidemic, it was thought that increased sporulation efficiency accounted for the enhanced transmission seen in hospitals, at least in part [33], however this has been disputed [32].

**a**. The robust properties of the spore are due to its multi-layered structure, with each layer contributing to the overall resilience [25]. The dense core is dehydrated due to the presence of up to 25% Ca-DPA, and the DNA is bound to and protected by the small acid-soluble proteins. Surrounding the core is an extremely impermeable inner membrane and the germ (primordial) cell wall. This thin layer of peptidoglycan has the same composition as in vegetative cells – and will become the nascent cell wall during germination. Around the germ cell wall is a much thicker layer of peptidoglycan, the cortex. Within the cortex peptidoglycan approximately 25% of the N-acetylmuramic acid moieties are modified to muramic-δ-lactam, and there are few crosslinks between adjacent N-acetylmuramic acid-N-acetylglucosamine polymers [26]. This results in a much more flexible peptidoglycan structure, with a distinct chemical signature that allows specific degradation during germination, without risk of compromising the germ cell wall. The cortex is surrounded by a second membrane, derived from the mother cell during engulfment, and then finally, the protein coat. The coat is a lamellar structure consisting of a large number of often highly crosslinked proteins. The outermost layers of the coat in *C. difficile* appear less organized, with an amorphous structure, and vary in thickness. This layer has been described as exosporium but does not appear to have the same loose-attachment and hexameric organization seen in other spore-formers [27]. Some of these structures can be seen in the transmission electron micrograph of a negative-stained, thin-sectioned spore on the right. **b**. When conditions are favorable, a *C. difficile* cell will normally be divided by binary fission. However, when environment conditions are less than ideal, most likely due to nutrient limitation, the cell can enter the sporulation pathway instead. Upon initiation of sporulation, the cell first undergoes asymmetric septation, producing the mother cell compartment and smaller forespore. A copy of the genome is transferred into the nascent spore, and the forespore is then engulfed by the mother cell - a phagocytosis-like event that results in an immature spore, bounded by two membranes, free in the cytoplasm of the mother cell. The spore then undergoes a maturation process whereby the DNA is compacted by the small acid-soluble proteins, Ca-DPA is synthesized, the core is dehydrated, and cortex and protein coats are synthesized. The final mature spore is released by lysis of the mother cell.

### Sporulation

Given the pivotal role the spore plays in disease transmission, it is not surprising that this has been an area of intense research in recent years. Sporulation begins with an asymmetric septation event that separates a larger mother cell from the smaller forespore compartment (Figure 1(b)). The mother cell then engulfs the forespore, resulting in an immature double-membraned prespore contained within the mother cell cytoplasm. A thick layer of cortex peptidoglycan is then synthesized between the two spore membranes but exterior to the existing primordial cell wall, and proteinaceous coat layers are assembled on the outer surface. The core contains a high concentration of dipicolinic acid coordinated to calcium ions (Ca-DPA) which functions to dehydrate the spore and protects the DNA from heat-induced damage [34–36]. The DNA is further protected from UV damage by small acid-soluble proteins (SASPs) [37]. Upon completion of synthesis of the cortex and coat layers the now mature spore is released by lysis of the mother cell. We will not attempt to exhaustively summarize the molecular basis of sporulation here, as this is already the subject of many excellent reviews in recent years [38,39].

Sporulation in *C. difficile* has many parallels with the well-studied Bacilli. However, this complex cell differentiation pathway has diverged significantly since the last common ancestor, likely over 2 billion years ago [40; 41], so caution must be exercised in extrapolating protein function based on homology, for even conserved components. The master regulator Spo0A and the subsequent sigma factor cascade is largely conserved, although with some difference in temporal regulation and mechanisms of sigma factor activation [42]. The signaling events upstream of Spo0A are not conserved and, despite the identification of several regulators that feed into *C. difficile* Spo0A expression, it is not yet clear how its activation is controlled [43–45]. Regulation of sporulation involves integration of multiple environmental and nutritional cues. Sporulation is very sensitive to environmental pH, with production of viable spores reduced in low pH, albeit with strain–strain differences observed [46]. The nutritional status of the cell is sensed via the catabolite control protein CcpA and CodY [45,47]. CcpA regulates approximately 9% of all *C. difficile* genes, ensuring a broad transcriptional response to glucose availability, and directly represses the expression of both Spo0A and the first forespore-specific sigma factor SigF [47]. CodY has a similarly broad role in gene regulation in response to nutrient status, sensing the availability of branched chain amino acids and GTP [45]. Expression of Spo0A and the first mother cell-specific sigma factor SigE is repressed by CodY and this repression is relieved under nutrient limitation. However, rather than directly regulating the *spo0A* gene as in *Bacillus* spp., in *C. difficile* CodY appears to act indirectly via the SinRI regulatory system. In addition to these well-characterized regulatory systems with parallels in other spore-forming Firmicutes, several Clostridia-specific regulators have also been described. The RNPP family regulator RstA has been shown to have pleiotropic roles in *C. difficile* gene regulation including activation of sporulation [48], via effects on the sigma factor cascade downstream of Spo0A. The signal sensed by RstA is currently unknown, although this family of proteins are often regulated by quorum sensing systems and can require direct binding to the autoinducing peptide [49]. Interestingly, sporulation is also subject to epigenetic regulation via the type II 6 mA methyltransferase CamA [50; 51]. Inactivation of *camA* and loss of the associated 6 mA DNA modification reduced sporulation by approximately 50%. Although the exact mechanism of this sporulation defect is unclear, it appears to halt sporulation following asymmetric septation and the expression of a large number of genes with putative roles in sporulation are affected. These pleiotropic effects are not unprecedented. We have previously shown that transposon mediated disruption of 798 individual genes in strain R20291 has a significant impact on sporulation efficiency, many of which with putative roles in gene regulation, including 53 annotated as “regulator” and 21 genes encoding parts of two component systems [50]. It is clear that much of the complex process of sporulation regulation remains to be elucidated.

### Germination

Once ingested, spores can readily survive the incredibly harsh low pH conditions of the stomach and, upon transit into the duodenum, begin to germinate back to vegetative cells that can colonize and proliferate in the colon. Germination is initiated in response to germinants, chemical signals that indicate the spore is in an environment that is conducive to vegetative cell survival and growth. In the case of *C. difficile*, the major germinant is the bile acid taurocholate [52]. Bile acids are surfactants that are produced in the liver from cholesterol, stored in the gallbladder and then secreted into the duodenum in response to food intake [53], where they emulsify dietary fats, aiding in their absorption and in the uptake of fat-soluble micronutrients [54]. Taurocholate is detected by the pseudoprotease CspC, which induces a signal cascade via CsbB to activate the cortex lytic hydrolase SleC that initiates germination [55]. Degradation of the cortex precedes Ca-DPA release [56], and the concomitant rehydration of the core allows metabolism to resume. Primary bile acids (those synthesized by the liver) are subject to chemical modification and degradation by members of the intestinal microbiota, generating the so-called secondary bile acids, with profound impacts on *C. difficile* germination and colonization (reviewed in [57]. Some secondary bile acids, including cholate, also act as *C. difficile* germinants while others, such as chenodeoxycholate, appear to act as direct inhibitors. Interestingly, deoxycholate can act as a *C. difficile* germinant but then inhibits outgrowth [52]. This cascade of bile acid metabolism likely represents one of the fundamental mechanisms of microbiota-bestowed colonization resistance [58]. Once induced, germination proceeds rapidly, with the new vegetative cells undergoing their first round of cell division within approximately 90–180 min [59]. Toxin production by these vegetative cells leads to the commonly seen symptoms of disease (see below), while subsequent rounds of sporulation generate a subpopulation of spores that ensures onward transmission to new hosts [28]. Spores also provide a reservoir of surviving viable bacteria that can lead to recurrent infection upon antibiotic cessation [60]. It was long assumed that this phenomenon was solely due to the intrinsic resistance of spores to the antibiotics that are commonly used to treat *C. difficile* infection. However, it has recently been reported that spores can enter intestinal epithelial cells, in an active process that involves BclA3 on the surface of the spores and host fibronectin and vitronectin and their cognate integrin receptors α_5_β_1_ and α_v_β_1_ [61]. This surprising observation hints at a previously unexpected *C. difficile* reservoir in which spores are shielded from germinants, allowing later reseeding of the colon should conditions be conducive for colonization.

## Virulence factors

Clinical presentation of CDI is influenced by a range of *C. difficile* virulence factors, including production of various toxins and surface proteins (Table 1). Primarily, pathogenesis is driven by the activity of toxins A and B, encoded within the pathogenicity locus (PaLoc). These toxins are internalized by gut epithelial cells, where they glucosylate small Rho proteins, resulting in cell death and loss of intestinal barrier function [62; 63]. Symptoms are further exacerbated by the host immune response, which involves an acute intestinal inflammatory response and neutrophil infiltration, further damaging the epithelia [64].

**Table 1.** Virulence factors of *C. difficile*.

Virulence Factor	Function/Evidence	References
Toxin A *(tcdA*)	Inactivate Rho GTPases. Disrupts the cytoskeleton resulting in disruption of tight junctions and loss of intestinal barrier function.	(Barth et al., 2001; Egerer et al., 2009; Gerhard et al., n.d.; Jank et al., 2007; Just et al., 1995; Madan and Petri, 2012; Oezguen et al., 2012; Papatheodorou et al., 2010; Qa’Dan et al., 2000)
Toxin B (*tcdB*)	Inactivate Rho GTPases. Disrupts the cytoskeleton resulting in disruption of tight junctions and loss of intestinal barrier function. Huge diversity of subtypes, undergoes accelerated evolution.	(Barth et al., 2001; Egerer et al., 2009; Gerhard et al., n.d.; Jank et al., 2007; Just et al., 1995; Madan and Petri, 2012; Oezguen et al., 2012; Papatheodorou et al., 2010; Qa’Dan et al., 2000; Shen et al., 2020)
*C. difficile* binary toxin (CDT)	ADP-ribosyltransferase which causes depolymerisation of the actin cytoskeleton (leading to loss of barrier function and disruption of tight junctions) and microtubule protrusions (leading to increased *C. difficile* adherence).	(Aktories et al., 2011; Gerding et al., 2014; Hemmasi et al., 2015; Papatheodorou et al., 2010; Schwan et al., 2009)
SlpA	Major S-layer constituent. S-layer null strain avirulent in hamster model, and more susceptible to lysozyme and immune effectors. Mutants making more porous S-layer display increased lysozyme sensitivity.	(Calabi et al., 2002; Kirk et al., 2017; Lanzoni-Mangutchi et al., 2022; Merrigan et al., 2013)
Cwp2	Implicated in adhesion. Dominant antigen in patient sera.	(Bradshaw *et al*., 2017)
Cwp84	Required for normal S-layer production. Dominant antigen in patient sera. However, mutants fully virulent in hamster models.	(Wright *et al*., n.d.; Kirby *et al*., 2009)
Cwp66	Implicated in adhesion and stress tolerance.	(Waligora *et al*., 2001; Zhou *et al*., 2022)
Cwp19	Transglycosylase involved in autolysis, resulting in toxin release.	(Wydau-Dematteis *et al*., 2018)
Cwp22	Peptidoglycan cross-linking enzyme (L,D-transpeptidase). Supports cell wall integrity. Mutation reduced toxin production, increased cell permeability and autolysis, and reduced adherence.	(Peltier et al., 2011; Zhu et al., 2019)
CwpV	Large phase-variable CWP. Displays auto-aggregative properties. Putatively involved in colonisation and biofilm *in vivo*. Confers resistance to some bacteriophage.	(Lawley et al., 2009; Reynolds *et al*., 2011; Sekulovic *et al*., 2015)
CD2831	Collagen binding protein involved in adhesion, biofilm formation and immune evasion.	(Arato *et al*., 2019)
CpbA	Involved in adherence through enhancing collagen interaction and extracellular matrix adherence.	(Tulli *et al*., 2013)
*Broader virulence traits*		
Lysozyme resistance	Resistance to hydrolysis via lysozyme due to σV activation of PgdA and PdaV. S-layer provides barrier protection. Required for successful pathogenesis in hamster models.	(Callewaert and Michiels, 2010; Fagan et al., 2009; Ho *et al*., 2014; Kaus *et al*., 2020; Lanzoni-Mangutchi *et al*., 2022)
Biofilm	Contributes to antimicrobial resistance, resistance to oxygen stress, persistence and recurrence of CDI.	(Bordeleau et al., 2014; Ðapa et al., 2012; Dawson *et al*., 2012; Frost et al., 2021; Poquet et al., 2018; Semenyuk et al., 2015; Soavelomandroso et al., 2017)
Spore formation	Essential for transmission of *C. difficile* and resistance to environmental stressors, such as oxygen, heat and UV damage. Enables disease persistence. Increased sporulation efficiency possibly increases disease transmission.	(Burns et al., 2011; Donnelly et al., 2016; Fimlaid et al., 2013; Merrigan et al., 2013; Nerber and Sorg, 2021; Setlow, 2007, 2006)
*tcdC* truncation	Truncation thought to increase production of toxins A and B, associated with hypervirulence in ribotype 027 strains.	(Carter *et al*., 2011; Gerding *et al*., 2014; Warny et al., 2005)

### The PaLoc

**Figure 2.**
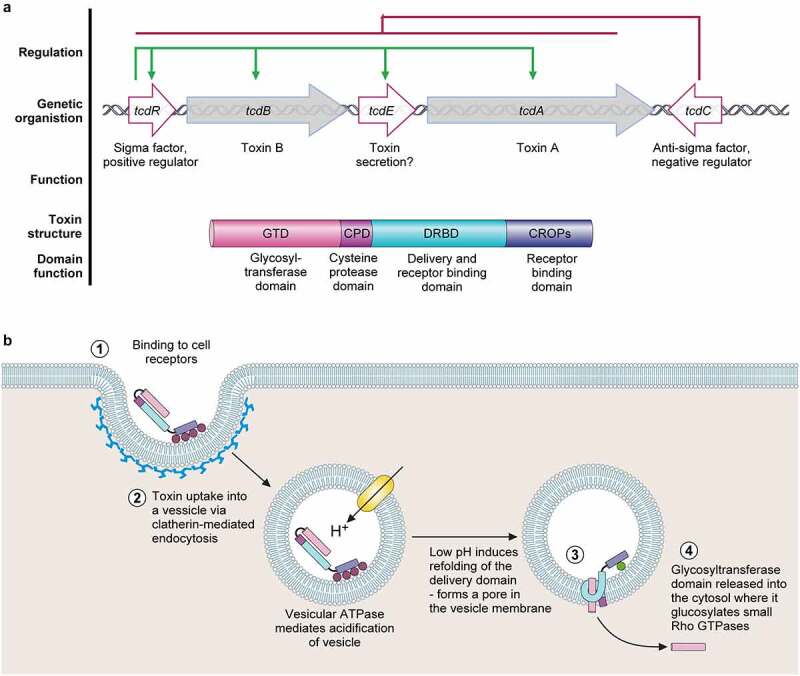
The pathogenicity locus and toxin mode of action. .

The *C. difficile* PaLoc spans a 19.6 kb region, with a typically highly conserved genomic localization and organization, and encodes 5 proteins involved in toxin-mediated pathogenesis (Figure 2(a)). This locus is finely regulated on multiple levels. First by environmental factors – toxin production is suppressed in nutritional excess, and transcribed during stationary phase or nutrient limitation [66]. Regulation also occurs at the population level – a recent visualization utilizing a dual-transcriptional reporter system demonstrated expression of toxin and sporulation genes rarely overlap, and solid growth results in subpopulations expressing either toxin (virulence) or sporulation (transmission) genes [67]. The 5 genes in the PaLoc include *tcdA* and *tcdB*, encoding toxins A and B, respectively; and *tcdR, tcdE* and *tcdC*. TcdR is an alternative sigma factor and likely positive regulator of toxin production, since purified *C. difficile* RNA polymerase was unable to bind to the *tcd* promoter regions in the absence of TcdR, and interaction of TcdR with the RNA polymerase holoenzyme allowed transcriptional activation [68; 69]. TcdR also activates its own promoter, in a positive feedback loop, allowing regulation of the entire PaLoc operon. TcdC is thought to be an anti-sigma factor involved in modulating toxin expression through sequestration of TcdR [70], however this requires further classification. The exact function of TcdE has previously been controversial, however it seems likely that this holin-like protein is involved in toxin secretion – as recently demonstrated in clinical strains [69,71]. Holins are membrane proteins, commonly encoded by double-stranded DNA phage, which are required for host cell lysis. The *tcdE* open reading frame contains three translational start sites resulting in TcdE isoforms of three different sizes. The involvement of combinations of these isoforms in both toxin release and cell death was demonstrated in the hypervirulent strain R20291 [68].

**a**. The pathogenicity locus (PaLoc) is composed of 5 genes: *tcdA* and *tcdB*, encoding toxins A and B respectively; *tcdR*, encoding an alternative sigma factor and likely positive regulator of the PaLoc (regulation shown above in green); *tcdE*, encoding a holin-like protein putatively involved in toxin secretion; and *tcdC*, an anti-sigma factor and negative regulator of the PaLoc genes (regulation shown above in red). Toxins A and B both consist of a broadly similar four-domain structure. At the N-terminal, the glucosyltransferase domain (GTD) is the active toxin moiety which inactivates members of the Rho GTPase family. A cysteine protease domain is next to the GTD, and is involved in auto-processing and release of the GTD. The next domain, often called the Delivery and Receptor Binding Domain (DRBD), contains a hydrophobic region and is thought to be involved in translocation of the GTD from the lumen of endocytic vesicles into the host cell cytoplasm. The final C-terminal receptor-binding domain (also known as C-terminal combined repetitive oligopeptides (CROPS) domain) binds to a range of cellular receptors.**b**. Toxin mode of action [65]. The toxins bind to various cellular receptors via the C-terminal CROPs domain, triggering clathrin-dependent endocytosis (**1**) followed by acidification of the resulting vesicle (**2**). The drop in pH triggers a conformational change in the delivery domain which inserts into, and forms a pore in, the vesicle membrane, through which the GTD transits into the host cytoplasm (**3**). The GTD is then released by a cleavage event mediated by the cysteine protease domain, in a process that is dependent on host inositol hexakisphosphate (**4**). The GTD is then able to glucosylate and inactive members of the small Rho GTPase family, including Rho, Rac, and Cdc42. Inactivation of Rho GTPases results in multi-level cellular disruption, including dysregulation of actin depolymerization, which causes disruption of tight junctions and loss of intestinal barrier function, induction of proinflammatory cytokines and activation of programmed cell death.

Toxins A (TcdA) and B (TcdB) consist of a broadly similar four-domain structure and are highly similar, with 47% amino acid identity, suggestive of an originating gene duplication event (Figure 2(a)). The N-terminal consists of a glucosyltransferase domain (GTD), next to which is a small cysteine protease domain, involved in auto-processing for the release of the GTD [72]. The next domain, often called the Delivery and Receptor Binding Domain (DRBD), contains a hydrophobic region and is thought to be involved in translocation of the GTD from the lumen of endocytic vesicles into the host cell cytoplasm. The C-terminal receptor-binding domain (also known as C-terminal combined repetitive oligopeptides (CROPS) domain) can bind to a range of carbohydrates, likely facilitating toxin binding to the cell surface [73].

Binding and internalization are essential prerequisites of *C. difficile* toxin-mediated pathogenesis. Despite their structural similarities, the binding repertoires of TcdA and TcdB are independent from one another. Early studies of TcdA receptors showed TcdA bound carbohydrate domains on the glucosidase enzyme sucrase-isomaltase [74], however this is not expressed in the colonic epithelium. Glycoprotein 96 was also identified to bind TcdA [75], however resides mainly in the endoplasmic reticulum, and therefore is unlikely to be the primary TcdA receptor [76]. More recently, CRISPR-Cas9 screens found that low-density lipoprotein receptors and sulfated glycosaminoglycans bound TcdA. Sulfated glycosaminoglycans were proposed as the major receptors, and were shown to be abundant in the colonic epithelium [76]. Although exploring binding interactions is essential to identify putative receptors, a recent study showed the importance of further characterization of such interactions. Low Density Lipoprotein Receptor-Related Protein-1 was proposed to serve as an endocytic receptor for TcdA, an important advancement, since TcdA must be internalized in order to act [77].

TcdB was shown to enter a variety of cell lines, indicating the existence of either multiple, or widely expressed, receptors [78]. The first recognized TcdB receptor was chondroitin sulfate proteoglycan 4 (CSPG4), a highly conserved protein, identified through shRNAmir library screening [79]. Cryo-EM structures of CSPG4-TcdB indicate binding is mediated through autoprocessing and delivery domains [80; 81]. However, CSPG4 receptors are abundantly expressed on subepithelial myofibroblasts, and not in the colonic epithelium, suggesting CSPG4 is not the dominant TcdB receptor [82]. Disruption of poliovirus receptor-like 3 (PVRL3) resulted in cells which were resistant to TcdB, leading to identification of a further TcdB binding partner [83]. PVRL3 is highly expressed in the colonic epithelia, however its role in TcdB-mediated pathogenesis has been disputed [84], and its contribution to infection remains unclear. More recently, the Frizzled receptors 1, 2, and 7 have been described as physiologically relevant binding partners of TcdB, due to their expression in the colonic epithelium [84]. Frizzled receptors interact with TcdB in a CROPS-independent manor, the crystal structure of which has recently been characterized [85]. Finally, clade 2 *C. difficile*, which includes hypervirulent 027 strains, express TcdB2 and TcdB4 – variants of TcdB which bind to distinct receptors. One such receptor, binding TcdB4, is tissue factor pathway inhibitor (TFPI) [86]. TFPI is expressed in the colonic crypt, and is therefore physiologically relevant.

The proposed mode of action, known as the ABCD model (activity (A), binding (B), cutting (C), delivery (D)), is similar between both TcdA and TcdB (Figure 2(b)) [65]. Here, TcdA and TcdB bind to cellular receptors as described above. Once bound, the toxins undergo endocytosis, through a clathrin-dynamin-dependent pathway [87]. Subsequent acidification of the endosome results in a conformational change of the toxin, allowing membrane insertion and channel formation [88; 89]. In the cytosol, the toxins undergo a further change, induced by the host virulence cofactor inositol hexakisphosphate. This allows activation of the toxin cysteine protease domain, and results in toxin autocleavage at a position between the cysteine protease and glucosyltransferase domains, releasing the glucosyltransferase domain into the cytosol [90;91]. Despite TcdB being able to induce cellular toxicity independent of the GTD, recent evidence suggests glucosyltransferase activity is still key for disease pathogenesis [92]. In the cytosol, the GTD is able to inactivate members of the Rho GTPase family, including Rho, Rac and Cdc42, via transfer of glucose from UDP-glucose to these proteins at a conserved threonine residue [93; 94]. Since Rho GTPases control pleiotropic signal transduction pathways, disruption to the host cell is widespread. Most notably, dysregulation of actin depolymerisation leads to disruption of the cytoskeleton, resulting in cell rounding, apoptosis, disruption of tight junctions and loss of intestinal barrier function [95; 96]. In fact, TcdA and TcdB are capable of causing both type I (apoptosis) and type III (necrosis) programmed cell death [97]. Changes in Rho GTPase function also evoke changes in proinflammatory signalling pathways, resulting in the production of proinflammatory cytokines IL-1β, TNF-α, IL-8. This, coupled with the subsequent influx of neutrophils, leads to further host tissue damage characteristic of CDI [98].

The relative contributions of TcdA and TcdB to virulence have commonly been disputed – historically, TcdA was accepted as the major virulence factor in *C. difficile*, owing to the significant immune response to TcdA, but not TcdB, observed in animal infection models [99]. However, the emergence of *C. difficile* clinical isolates producing only TcdB led to a re-evaluation of the roles of each toxin in disease [100]. Isogenic ribotype 027 toxin mutants demonstrated that both toxins contribute to fulminant disease in hamster models independently [101]. However, contending studies using a variety of animal models showed attenuated virulence in TcdA-producing isogenic strains, with full virulence in TcdB-producing strains [102]. Despite such discrepancies, TcdB is now widely accepted as the major *C. difficile* virulence factor, due to its involvement in invoking both local and systemic host damage, and activation of the host inflammatory response [102; 103]. Interestingly, a variety of toxin variants of TcdB, but not TcdA, have been reported. Owing to this, a recent global comparison of available TcdB sequences found huge diversity in this protein, allowing an 8-subtype classification. TcdB undergoes accelerated evolution, maximizing diversity and impacting pathogenicity and disease progression [104].

The location of *tcdC* at the end of the PaLoc, its divergent transcription and its inverse expression profile (high transcription during exponential, low transcription during stationary phase) compared with the rest of the PaLoc genes has led to its common association with PaLoc repression [70,105]. The role of TcdC as a modulator of toxin expression is abundantly apparent *in vivo* – mutations truncating TcdC are widespread in hypervirulent clinical isolates, and are commonly acknowledged to contribute to the high mortality of ribotype 027 strains [15]. However, the diverse genomic backgrounds of such clinical strains have made confirming this relationship difficult. In an attempt to combat this, analysis of isogenic *C. difficile* ribotype 027 strains revealed that mutation of *tcdC* led to hypervirulence, and complementation reduced virulence in hamster models [106]. Despite the wealth of functional evidence for TcdC as a negative regulator of toxin expression, mechanistic detail is still lacking. TcdC has been proposed to act as an anti-sigma factor, through interfering with the binding of RNAP to the *tcdA* promoter. The mechanism of this inhibition is unclear – TcdC may inhibit interaction of the TcdR sigma factor with RNAP, or prevent recognition of the *tcdA* promoter [70]. More recently, a detailed topological analysis of C-terminally tagged TcdC suggested an extracellular localization. This localization is discordant with previous work, suggesting TcdC may not act as an anti-sigma factor, and highlighting that the functional characterization of TcdC is far from complete [107].

### Binary toxin

Characterization of the hypervirulent ribotype 027 epidemic strain, first reported at the start of the millennium, showed a combination of factors putatively involved in increased virulence: high-level fluoroquinolone resistance, *tcdC* mutation leading to increased PaLoc expression, and possession of a further toxin – *C. difficile* binary toxin (CDT) [108]. CDT is an ADP-ribosylating toxin, composed of 2 proteins, the crystal structures of which have been reported recently [109; 110]. CDTa is an ADP-ribosyltransferase, the enzymatic component involved in modifying host cell actin; while CDTb is involved in binding to host cells and translocating CDTa to the host cytosol. CDT first binds to host cells via the lipolysis-stimulated lipoprotein receptor, present in host cell liver, kidney, small intestine and colon [111]. This binding is followed by accumulation of lipid rafts, oligomerization and induction of endocytosis [87,112]. In the resulting endosome, acidification triggers membrane insertion and pore formation by CDTb, allowing translocation of CDTa into the cytosol. Refolding of CDTa after translocation is mediated by host chaperones, including Hsp90 and Cyp40 [113]. CDTa then ADP-ribosylates cellular actin at Arg177, producing ADP-ribose and nicotinamide as biproducts. The modified actin is prevented from further polymerization due to the ADP-ribose moiety. Eventually, this leads to complete depolymerization of the actin cytoskeleton, resulting in phenotypes typical for toxins affecting the cytoskeleton, including loss of barrier function and disruption of tight junctions [114; 115]. However, CDT displays a multifaceted approach to host cell toxicity, since actin polymerization results in redistribution of the microtubule network. Microtubules are involved in a range of cellular processes, including intracellular transport, cell division and cilia formation [116]. CDT hijacks this network, resulting in formation of long cellular protrusions which increase *C. difficile* adherence to host cells, both *in vitro* and in mouse models [115,117]. Details of the mechanism of CDT are reviewed in detail elsewhere [15,114].

The regulation of CDT is distinct from, but entwined with, the PaLoc; since *cdtA* and *cdtB* are located on a separate 6.2 kb chromosomal region of the genome, known as CdtLoc. CdtLoc contains the two genes encoding CDT, and *cdtR* – a LytTR family orphan response regulator. CdtR is a positive regulator of both CDT and the PaLoc in hypervirulent strains [118]. Non-CDT producing *C. difficile* strains contain either a truncated version of the CdtLoc, or a 68 bp insertion sequence at this site [119].

The substantial contribution of CDT to *C. difficile* pathogenesis has become increasingly apparent through both clinical and experimental works. Several cases of patients with *C. difficile* infections, with the unusual toxinotype of TcdA and TcdB negative/CDT positive have been reported [120]. Although rare, these cases show CDT alone is capable of causing symptomatic infection. These observations have been confirmed experimentally – an isogenic TcdA/TcdB negative ribotype 027 *C. difficile* strain, expressing only CDT, caused disease phenotypes in hamster models [101]. However, such phenotypes were different to typical CDI, with symptoms reminiscent of small intestine involvement. Further analysis of the role of CDT in conjunction with the PaLoc showed CDT contributes to increased virulence and disease severity, with mouse models exhibiting increased weight loss and higher mortality compared to a CDT-negative strain [121]. This also highlighted the role of CDT in activating the inflammatory response, with elevated IL-6 cytokine levels observed in mouse models, compared to CDT-negative *C. difficile*. CDT also induces inflammation via the Toll-like receptor 2-dependent pathway, and suppresses the protective host eosinophil response [121]. Recent evidence has also implicated CDT in the activation of cytotoxic responses in human mucosal-associated invariant T-cells, leading to further aggravation of the pro-inflammatory response [122]. Collectively, these studies suggest CDT is an important virulence factor in *C. difficile* pathogenesis, particularly in hypervirulent strains.

### Surface proteins: S-layer

*C.difficile* surface proteins are a group of important virulence factors which support *C. difficile* colonization through adherence to the gut epithelium, activation of the host immune response, and other aspects of pathogenesis. The S-layer is an evolutionary conserved paracrystalline array of protein which envelops the cell, and is ubiquitous among *C. difficile* strains [123; 124]. It is one of the most metabolically expensive components of the cell, consuming a large percentage of the total cellular protein production [124]. Primarily, this layer is composed of two subunits – the low-molecular weight and high-molecular weight S-layer proteins, which are derived from the post-translational cleavage of a single precursor (SlpA) [125]. These subunits form a heterodimer, which self-assembles to form the S-layer [126; 127]. Accompanying this core structure are 28 cell wall proteins (CWP), which comprise 5–20% of the S-layer and provide a range of additional functions [124].

The essentiality of SlpA, and thus the lack of available isogenic *slpA* mutants, has impeded the complete functional characterization of the S-layer. However, isolation and analysis of a spontaneous S-layer null strain has implicated the S-layer in sporulation, and resistance to innate immune effectors including lysozyme and LL-37 [128]. Collectively, these findings suggest the S-layer plays an important multifunctional role in successful pathogenesis. Further, the spontaneous S-layer null strain was avirulent in the acute hamster model of CDI, despite persistent colonization in the cecum and colon – suggesting the S-layer is a crucial virulence factor. Both *in vivo* and *in vitro*, toxin release of the S-layer null strain was markedly reduced compared to wildtype, associating the S-layer with toxin production, albeit via an unknown mechanism [128]. Strong and specific binding of S-layer proteins to human gastrointestinal tissue specimens have previously been reported, with the strongest binding on the surface epithelium lining the lumen [129]. Binding of the S-layer to HEp-2 cell lines has also been shown, and addition of anti-SlpA antisera led to a 50% reduction in binding to monolayers of multiple *C. difficile* strains – collectively suggesting an important role in colonization and disease establishment [130]. The apparent contradiction between these observations and the lack of a colonization defect seen with the S-layer null strain in hamsters suggests that there are aspects of *C. difficile* ecology in the gut that we do not fully understand.

### Surface proteins: CWPs

The 28 members of the CWP family are defined by the presence of three tandem copies of the cell wall-binding 2 domain (PF04122), with many also having additional individual domains conferring function [124]. Twelve members of the family are encoded within the CWP gene cluster, a 36.6 kb region including *slpA*, and related genes, and adjacent to a cluster of genes thought to be involved in the synthesis of the cell wall polysaccharide PS-II [131]. The remaining CWP genes are distributed throughout the genome [132].

Many CWPs are associated with pathogenesis, and are often highly immunogenic. Antibodies to a range of CWPs, most prominently Cwp2 and Cwp84, were found in convalescent patient sera [133]. Such CWPs are therefore likely to be expressed and surface accessible during colonization and/or pathogenesis. Since Cwp2 is the most highly expressed constitutive CWP, and is highly immunogenic, it is perhaps surprising that a *cwp2* knockout displayed no defects in growth, sporulation, or virulence in hamster models [134]. However, a significant reduction in adherence to Caco-2 cells suggests this protein functions mainly as an adhesin. Similarly, the immunogenicity of Cwp84 is not concordant with virulence – despite being highly conserved, and responsible for SlpA cleavage, *cwp84* mutants were fully virulent in hamster models [135]. Cwp66 is a 66 kDa protein, containing the typical cell wall binding domains, and an additional domain of unknown function. This protein was the first *C. difficile* classified adhesin, since antibodies to Cwp66 reduced cellular adherence [136]. Recently, molecular characterisation of a Δ*cwp66* mutant implicated this surface protein in adhesion, motility and stress tolerance [137]. Further, transcriptomic analysis of Δ*cwp66* suggested a wider cellular involvement of the protein in antimicrobial resistance and metabolism – signifying a multifactorial role in pathogenesis [137]. Cwp22 is an L,D-transpeptidase, a peptidoglycan cross-linking enzyme, that contributes to multiple aspects of pathogenesis [138; 139]. Mutation of *cwp22* led to reduced toxin production, and increased cell permeability and autolysis, as well as reduced cellular adherence. Further, *cwp22* mutants displayed reduced fitness compared to WT in mice, collectively suggesting that this protein plays important roles in cell envelope integrity and pathogenesis [139]. A further CWP linked to the *C. difficile* toxins, Cwp19, has been identified as a transglycosylase, contributing to *C. difficile* pathogenesis through autolysis, resulting in toxin release in specific environmental conditions [140]. CwpV is the largest protein in the CWP family, and exhibits phase-variable expression [141]. As with SlpA, CwpV is subject to post-translational cleavage followed by stable interaction between the resulting cleavage products [142]. The CwpV-specific C-terminal domain consists of a series of repeats that are highly variable between *C. difficile* strains, with five distinct repeat types identified to date [143]. Functional characterization has demonstrated that CwpV can contribute to auto-aggregative cell–cell interactions and as such, is postulated to be involved in colonization and the biofilm-like growth that is observed *in vivo* [60].

### Surface proteins: Collagen-binding proteins

As with all pathogenic bacteria, multiple complex mechanisms allow fine-tuned host interactions and immune evasion. One such mechanism, binding to the host extracellular matrix, has recently been described in *C. difficile*. CD2831 is a collagen binding protein, which further promotes adhesion and biofilm formation [144]. CD2831 also enhances immune evasion, through binding the collagen-like domain of C1q of the complement pathway, modulating the classical immune response [144]. Similarly, *C. difficile* produces an additional collagen-binding surface protein, CbpA. Despite the *cpbA* knockout being indistinguishable from its respective WT during immobilized collagen V binding assays, this protein enhances collagen interaction and extracellular matrix adherence, demonstrating the large redundancy involved in host interaction and pathogenesis [145].

### Lysozyme resistance

Lysozyme is a ubiquitous and highly conserved antimicrobial protein involved in the innate immune response. This antimicrobial targets the bacterial cell wall, cleaving the β(1–4) bond between N-acetylglucosamine and N-acetylmuramic acid of peptidoglycan [146]. *C. difficile* is highly resistant to cell wall hydrolysis via lysozyme, due to a combination of important virulence factors. The *C. difficile* S-layer clearly provides some protection against lysozyme, since an S-layer null mutant becomes sensitive to physiological concentrations of the enzyme [128]. Deletion of domain 2 of the low-molecular weight S-layer protein [126] increased the apparent permeability of the assembled S-layer and rendered *C. difficile* susceptible to lysozyme, suggesting that a steric barrier function contributes to S-layer-mediated resistance to innate immune effectors [147]. In addition to this barrier protection, *C. difficile* also has a further inducible resistance system that is controlled by the extracytoplasmic sigma factor σV [148]. σV is activated upon lysozyme detection, resulting in expression of peptidoglycan deacetylases PgdA and PdaV [148]. Classical microbiology has shown the synergistic effects of these two proteins in their contribution to lysozyme resistance – deletion of a single protein resulted in small reductions in resistance, whereas deletion of both proteins simultaneously resulted in 1000× reduction in resistance, through almost complete loss of peptidoglycan deacetylation [149]. Deacetylation is an effective method of lysozyme resistance, since interaction between the activate site of lysozyme, and acetyl groups on peptidoglycan enables efficient hydrolysis [150]. Moreover, σV is required for successful pathogenesis in hamster models, demonstrating the importance of lysozyme resistance mechanisms as virulence factors [148].

### Biofilm

The clear contribution of biofilms to both virulence and antimicrobial resistance, along with improved tools and novel study methods, has led to the emergence of biofilms as a “hot topic” in microbiology over the last decade. Despite this, little is known about the formation, regulation and maintenance of *C. difficile* biofilms. *C. difficile* forms part of the healthy, multi-species biofilm during asymptomatic carriage [151]. However, it has been hypothesized that biofilms may, in fact, also play a role in the persistence and recurrence of CDI [152]. Despite the picture being far from complete, multiple factors have been associated with biofilm formation and regulation in *C. difficile*. Of note, the sporulation master-regulator Spo0A is associated with regulation of biofilm formation, with mutants exhibiting significantly reduced biofilm [153]. The second messenger c-di-GMP, known to regulate the switch from motile single-cellular to multicellular formations in gram-negative organisms has also been implicated in *C. difficile* biofilm formation [154]. Increased c-di-GMP reduced flagellar motility and upregulated type 4 pili – increasing cell aggregation [154; 155]. Multiple other genes have also been implicated in biofilm formation – *cwp84* mutants displayed a severe defect in biofilm formation, as did mutants lacking the quorum sensing regulator LuxS [153]. The biofilm lifestyle is thought to be dampened through expression of DnaK, a stress response protein, since alterations to *dnaK* result in stronger biofilms [156]. Likewise, LexA, the global transcriptional repressor and inducer of the SOS response, also reduces biofilm formation, with Δ*lexA* mutants showing reduced sporulation, motility, and increased biofilm formation [157]. The complex regulation of biofilm formation is therefore clearly mediated in part by both stress and quorum sensing. A detailed review of *C. difficile* biofilm regulation can be found here [158].

More broadly, the contribution of biofilm to *C. difficile-*host interactions has been explored through confocal laser scanning microscopy in mouse models. In a mono-associated mouse model, which simplifies analysis of pathogen–host interactions without competition from the microbiota, *C. difficile* was found to produce a 3D biofilm associated with the mucus layer [159]. Cells were entrapped in a glycan matrix, composed largely of the bacterial polysaccharide PS-II. To attain a more realistic view of the role of commensal *C. difficile* biofilm in relation to the host gut, 16S rRNA analysis identified *C. difficile* as a minor part of the complex multispecies host biofilm, composed of *Bacteroidetes* and *Firmicutes* [160]. *C. difficile* biofilms may be important for virulence, since they enhance survival through improved resistance to antibiotics and oxygen stress [161]. However, more in-depth studies of the dynamics of such *in vivo* biofilms are needed to fully understand the contribution of this lifestyle to pathogenesis.

## The contribution of antibiotic resistance to pathogenesis

**Figure 3.**
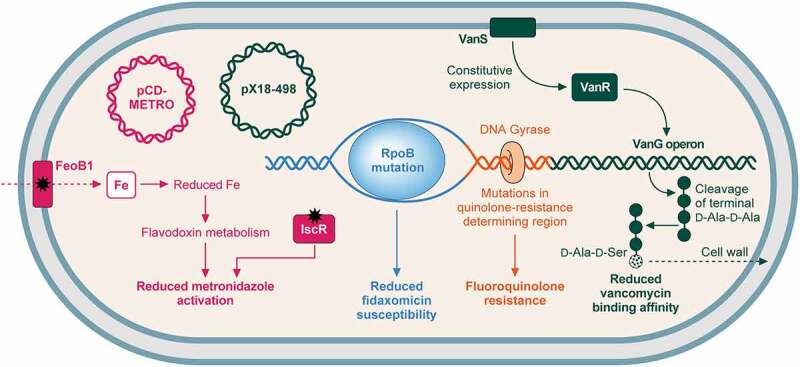
Mechanisms of resistance to commonly used antibiotics. .

One of the most important factors in *C. difficile* pathogenesis is antibiotic resistance (Figure 3). Prior exposure to antibiotics has long since been accepted as the primary risk factor for CDI, since increased abundance of *C. difficile* in the colon correlates with dysbiosis, most commonly caused through antibiotic exposure [60]. Being intrinsically highly resistant to a multitude of antibiotics further increases virulence, and significantly reduces treatment options. The major complication of CDI, recurrence, is also attributed to exacerbation of gut dysbiosis due to antibiotic treatment. Thus, antibiotic resistance allows colonization, avoidance of clearance, persistence, and recurrence – impacting all aspects of infection.

Mechanisms of *C. difficile* resistance to antibiotics commonly used to treat CDI (vancomycin, fidaxomicin, and metronidazole) and fluoroquinolones. (i) metronidazole (pink): resistance can be gained via the plasmid pCD-METRO [162]. Metronidazole resistance may also be gained through mutation of either FeoB1, which reduces intracellular iron, reducing flavodoxin metabolism and metronidazole activation; or IscR, which also reduces metronidazole activation [163]. (ii) fidaxomicin (blue): mutations in RpoB reduce fidaxomicin susceptibility [164]. (iii) vancomycin (green): Mutations in *vanSR* two-component system allow constitutive expression of the *vanG*-like operon, which aids vancomycin resistance through replacement of the terminal d-alanine in peptidoglycan pentapeptide sidechains with d-serine, reducing vancomycin binding affinity [104,165]. Plasmid pX18-498 has also recently been associated with resistance, although the mechanism is not understood [166]. (iv) fluoroquinolones (orange): mutations in the genes encoding DNA gyrase, particularly *gyrA* results in fluoroquinolone resistance [167].

A multitude of evidence supports antibiotics as the major risk factors for CDI. The mechanism of microbiome-associated colonization resistance is far from clear, but is likely a multi-faceted phenomenon involving competition for nutrients, immune modulation, and production of harmful metabolites [168]. The best understood factor is the impact on bile acid metabolism described above, and in particular, the conversion of deconjugated primary bile acids to deoxycholate and lithocholate by members of the microbiome with 7α-dehydroxylase activity [169]. Treatment with antibiotics, either prophylactically or for another infection, causes severe and unpredictable disruption to the resident microbiome. Changes in diversity and relative abundance of species within the microbiome reduces colonisation resistance in the colon, allowing *C. difficile* to colonize and flourish [60]. Supporting this, a wealth of clinical evidence links antibiotic exposure to CDI: retrospective cohort studies have implicated dose, number of antibiotics used, and days of antibiotic exposure with CDI, with risk increasing in a dose-dependent manner [170]. Further, a striking recent study suggested that odds of infection increased by 12.8% with every day of antibiotic therapy – however this was dependent on both the antibiotic used, and route of administration [171]. Although many antimicrobials are associated with CDI, risk is most highly associated with broad-spectrum antibiotics, including cephalosporins, carbapenems, fluoroquinolones, and clindamycin [171]. In mouse models, clindamycin reduced microbiome diversity by 90% for 28 days. This increased CDI mortality and led to colonic inflammation even in recovering mice – suggesting antibiotic exposure increases not only the risk but severity of CDI [172]. Of course, recurrence – either through relapse or reinfection – is also highly associated with antibiotic use [173]. In pediatric recurrent CDI patients, antibiotic exposure and recent surgery were significant recurrence risk factors [174]. Other clinical studies report similar outcomes, with antibiotics, and previous use of fluoroquinolones, being independent risk factors for recurrence [9]. Therefore, antibiotic use undoubtedly has a large impact on the ability of *C. difficile* to act as an opportunistic pathogen.

The success of *C. difficile* as a pathogen is inherently linked to its ability to resist antibiotics. The 4.29 Mb genome of *C. difficile* has demonstrated an extraordinary ability to gain resistance to a multitude of antibiotics, including aminoglycosides, tetracyclines, erythromycin, clindamycin, beta-lactams, and cephalosporins [175; 176]. This multidrug resistance was the driving force of the CDI epidemic at the start of the millennium, in addition to emergence of novel epidemic lineages, highlighting the importance of such factors in pathogenesis. Resistance to the macrolide-lincosamide-streptograminB (MLS_B_) family of antibiotics, encompassing erythromycin and clindamycin, is achieved through ribosomal methylation, and is gained via acquisition of transposons, such as Tn5398, containing *erm* genes [175,177]. *erm* encodes a 23S rRNA methylase, which modifies the 23S rRNA of the 50S ribosomal subunit, reducing drug binding affinity [178]. However, several *C. difficile* erythromycin-resistant strains have been identified which lack *erm* genes – suggesting the presence of yet uncharacterized alternative resistance mechanisms [179]. Tetracycline resistance is less widespread in *C. difficile*, however conjugative transposons have allowed transfer of *tet*M to certain strains, providing a mechanism of ribosome protection against tetracycline [180]. Perhaps, the most intriguing capability is that of fluoroquinolone resistance. Not unusually, resistance occurs via alterations to the DNA gyrase subunits, typically GyrA (Figure 3) [181]. However, the emergence of ribotype 027 was associated with widespread fluoroquinolone use, and the epidemic strains possessed recently acquired high-level fluoroquinolone resistance. Since antibiotics target essential cellular processes, resistance is often associated with large fitness burdens. However, competition analysis using mutations seen in *C. difficile* 027 clinical isolates found fluoroquinolone resistance did not lead fitness costs *in vitro*, suggesting that this property will persist in the species even in the context of improved fluoroquinolone stewardship [167].

## Resistance to antibiotics used to treat *C. difficile*

Of course, being resistant to a wealth of antibiotics poses two challenges: (i) the extensive resistance displayed greatly reduces treatment options for CDI, warranting the status of *C. difficile* as an urgent threat; and (ii) such treatment options are likely to be further limited through the high degree of adaptation and flexibility in the *C. difficile* genome. Until recently, three antibiotics were commonplace for the treatment of CDI. Metronidazole was typically the antibiotic of choice for mild-to-moderate CDI in first instance of infection, while vancomycin was reserved for severe and severe-complicated disease. Fidaxomicin – a narrow-spectrum antibiotic, effective against gram-positive anaerobes – was often overlooked due to higher cost, being significantly more expensive than metronidazole [182; 183]. In 2021, vancomycin became the NICE-recommended front-line antibiotic for CDI, replacing metronidazole as the first-instance treatment [184]. This move reflects both high metronidazole-related recurrence rates, and increasing reports of metronidazole resistance, but poses risks of its own in terms of increasing vancomycin selection pressures.

### Metronidazole

Metronidazole is a nitroimidazole antibiotic, effective against anaerobes via formation of unstable nitroimidazole anions, which, when converted into reactive intermediates, react with cellular components to form harmful adducts [163]. Alongside the recent emergence of various epidemic lineages, there has been an increase in metronidazole treatment failure [185]. Resistance in *C. difficile* was previously thought to be transient, however a recent explosion in research focussed on characterizing metronidazole resistance has led to the discovery of multiple heritable pathways to reduced susceptibility (Figure 3). One such mechanism involved a 7-kb plasmid, dubbed pCD-METRO, that increased resistance 25-fold, and conferred stable resistance to metronidazole [162]. Worryingly, pCD-METRO is thought to be horizontally transferrable, and is already internationally disseminated. However, the lack of universality of this mechanism implies the existence of multiple pathways to metronidazole resistance. Further clinical isolate studies found multiple SNPs in genes affecting iron utilization and electron transport – hinting at the molecular mechanism of resistance [186]. This mechanism was later uncovered using an evolutionary approach, which demonstrated the involvement of redox and iron homoeostasis genes, in a deterministic route to resistance [163]. The existence of multiple routes of resistance to what was once the first-instance treatment for CDI demonstrates how even antibiotics used to treat *C. difficile* can further contribute to pathogenesis through treatment failure and recurrence.

### Fidaxomicin

The current second-line antibiotic, fidaxomicin, acts to inhibit RNA-polymerase at a site distinct from rifamycin through binding to the DNA-template-RNA-polymerase complex prior to transcription initiation. This traps the complex in an “open clamp” position, preventing interaction with the −35 and −10 sequence [187; 188]. Despite the use of fidaxomicin being curtailed due to cost, it displays clear benefits to CDI treatment – its narrower-spectrum of activity results in reduced rates of recurrence compared to alternative treatments [62]. It is worrying, therefore, that resistance has recently been described (Figure 3). Clinical isolate Goe-91 was found to have mutations in *rpoB*, seen previously in laboratory studies [164]. However, this clinical isolate displayed no apparent fitness burden in terms of growth and sporulation [189]. Since fidaxomicin is already rarely used, emerging resistance casts doubts over the longevity of this CDI treatment.

### Vancomycin

Vancomycin is a glycopeptide antibiotic used for treatment of Gram-positive pathogens. Vancomycin impacts the cell wall biogenesis at multiple levels – binding the terminal D-ala-D-ala to prevent crosslinking of peptide chains by transpeptidases, whilst also binding and inhibiting the glycosyltransferase enzyme involved in polymerization of the NAM-NAG sugar backbone. Such activities have bactericidal action on cells through osmotic stress [190]. Despite being well-characterized in other species, and now predominantly used for CDI, vancomycin resistance in *C. difficile* has been poorly defined. That said, vancomycin resistance rates have increased substantially since 2012, correlating with an increased usage worldwide [191]. The whole-genome sequence of *C. difficile* published in 2006 revealed a *vanG*-like cluster, proposed to confer resistance through changing the terminal D-Ala-D-Ala residues to D-Ala-D-Ser, reducing drug binding affinity [165; 192]. Using an evolutionary approach, the two-component system *vanSR*, responsible for regulating the *vanG* operon, was shown to be constitutively expressed in isolates with reduced vancomycin susceptibility (Figure 3) [104]. Recent detection of vancomycin-resistant clinical isolates did not, however, identify mutations in this cluster, again suggesting multiple mechanisms of resistance, and demonstrating the frightening plasticity of the *C. difficile* genome [193]. 2021 also marked the first documentation of plasmid-mediated vancomycin resistance in *C. difficile*, through a broad-host-range and highly transferable plasmid. Plasmid p × 18–498 was associated with reduced vancomycin susceptibility *in vitro*, and more severe CDI *in vivo* in mouse models, highlighting the role of both resistance and plasmid carriage in *C. difficile* pathogenesis [166].

## Therapeutics: Current and future

The increasing threat of antimicrobial resistance, coupled with the diminishing number of available treatments has driven interest in both novel antimicrobials and alternative therapeutics for the treatment of CDI. Since the root of the problem lies with broad-spectrum antibiotics, new approaches aim to shift the archetypal *C. difficile* treatment to be more targeted and narrower spectrum, reducing further exacerbations of dysbiosis and risk of recurrence. Such treatments include faecal microbial transplantation (FMT), phage therapy, and narrow-spectrum antimicrobials.

### FMT

FMT involves administration of faeces from a healthy individual (heterologous), or from one’s own previously healthy microbiome (autologous) to restore the natural gut flora. This has gained popularity as a treatment for CDI over the last decade, however the procedure has yet to be standardized, and there have been reports of adverse events post-transplantation [194]. Typically, faeces can be delivered via colonoscopy, enema, nasogastric tube or oral capsules [195]. The virtues of FMT are well established, as both a standalone and combination therapy: one trial suggested clinical resolution following FMT was 92% [196], while another found FMT with vancomycin provided an 81% clinical resolution of CDI, compared to just 31% for vancomycin alone [197]. Moreover, the potential of FMT to treat the major complication, recurrent CDI, should not be forgotten – a recent study found a 68% success rate across complex patients with recurrent CDI alongside multiple co-morbidities and extended antibiotic use [198]. Despite intense effort in recent years, the underlying mechanism of FMT-mediated restoration of colonization resistance is still disputed but likely involves a combination of competition for resources, immune modulation and production of inhibitory metabolites. Intriguingly however, a very small trial of only 5 patients demonstrated a high rate of CDI resolution using a sterile fecal filtrate, hinting that resident bacteriophage could also be a contributory factor in the effectiveness of FMT [199].

Despite the clear effectiveness of FMT, its unconventional nature has limited public acceptance, and the lack of process standardization poses a worry to clinicians. Further, upon progression to pseudomembranous colitis, FMT has reduced efficacy and often requires repeat treatment [200]. There is also a question mark over manipulation of the microbiome – despite huge advancement in metagenomics, a complete understanding of the gut microbiome, and essential constituents, is lacking [201]. Most importantly, larger, randomized-controlled clinical trials are required to fully understand the efficacy and safety of FMT, since the nature of the therapy holds the risk of transferring pathogens to already-vulnerable patients [194]. Taken together, FMT provides a feasible alternative therapy for CDI, however there are many challenges to overcome before it becomes mainstream. A more refined approach to FMT is clearly desirable, and this is reflected in the array of new microbiome-based therapeutics in clinical development or already undergoing clinical trials for the treatment of CDI. Among these are those derived from donor feces, such as SER-109 from Seres Therapeutics, consisting of spores of mixed Firmicute species purified following ethanol treatment [202], and suspensions of defined bacterial communities grown in pure culture such as the 8-species VE303 from Vedanta Biosciences [203]. Both approaches show great promise and highlight the progression toward more targeted manipulation of the microbiome for therapeutic purposes.

### Phage therapy

Phage therapy involves the use of naturally-occurring bacteriophages to infect and lyse pathogenic bacteria [204]. This has been long-established as a potential therapeutic approach, especially at the coal face of the antimicrobial resistance crisis. The major benefit of using phage, as opposed to static antimicrobial agents, is the ability of phage to evolve. Much like with antibiotics, bacteria can, and have, evolved mechanisms of resistance to phage invasion [205; 206] – however unlike antibiotics, phage have evolved numerous ways to overcome such defences [207]. This continual evolutionary arms-race means phage therapies will not become obsolete [208]. Multiple phages infecting *C. difficile* have been identified, and increasing evidence suggests at least some of these are viable therapeutic agents [209], for example, ФCD27 was shown to reduce *C. difficile* growth and toxin levels in a batch fermentation model of CDI [210]. To date, no strictly lytic *C. difficile* phage have been identified, and all of the characterized temperate phage are double-strand DNA viruses, either contractile myoviruses or non-contractile siphoviruses [211]. Additionally, few *C. difficile* cell surface receptors have been identified, however the S-layer seems to be a common target [128; 212; 213; 214; 215]. Given the high degree of sequence variability seen in the S-layer [123], it is likely that a cocktail of phage would be required in an effective CDI therapeutic. One such combination of phages has been shown to cause complete *C. difficile* lysis *in vitro*, and to reduce disease symptoms and bacterial colonization in hamster models, suggesting targeted phage cocktails may be feasible treatment options [216]. More recently, this particular cocktail was further optimized to a combination of 4 phage, which showed complete *C. difficile* eradication in fermentation vessels [217].

R-type bacteriocins, phage tail-like particles that are structurally similar to the contractile myoviruses, have also been explored as potential therapeutic agents [128,218]. These have the benefit of bypassing many natural phage resistance systems, as they have no genome, but have the same host-range limitations as phage and present additional production challenges as they are not self-perpetuating. Genetic engineering could allow us to overcome many of the limitations of naturally occurring phage and phage tail-like particles. In recent years engineered phage have had some high-profile clinical successes, for example in the treatment of a recalcitrant *Mycobacterium abscessus* infection [219]. Similar approaches have been adopted for CDI, including altering the target spectrum of phage tail-like particles by swapping receptor binding proteins [128] and enhancement of killing, both *in vitro* and *in vivo*, by engineered reduction of lysogeny and redirection of the endogenous type 1-B CRISPR Cas system to target the host’s own genome [220]. This latter approach was the first demonstration that a *C. difficile* phage could be engineered to be lytic, albeit not completely, and showed that cargo DNA could be added to the phage genome with no apparent impact on the efficiency of infection or formation of progeny phage. As our understanding of both phage infection and host resistance improves it seems likely that engineered optimized phage will play an important role in the treatment of bacterial infections, particularly those such as CDI, where species-specificity is paramount.

As with all novel therapeutics, phage therapy does not come without limitations. While phage therapy is generally regarded as safe, and small human trials have not reported adverse effects, the possibility of harm to the patient cannot be ruled out [221]. Host inflammatory responses to phage have been reported in *in vivo* models, suggesting the possibility of adverse reactions which may worsen disease [222]. One possible therapeutic approach to avoid such downsides is to use engineered phage-derived biomolecules rather than whole phage [223], with phage endolysins emerging as a clear favorite in recent years [224–226]. Overall, the specificity, coupled with the ability to counter bacterial resistance, suggests great promise for phage treatment as a *C. difficile* therapeutic.

### Antibody therapies

Few novel *C. difficile* specific therapeutics have come to market in recent years. Fidaxomicin was approved by the FDA in 2011 and the human monoclonal antibody bezlotoxumab followed in 2016. Bezlotoxumab binds to two highly similar sites within the TcdB CROPs domain, thereby blocking binding of the toxin to carbohydrate receptors [227]. Interaction between the antibody and TcdB prevents intoxication and, in combination with an anti-TcdA monoclonal actoxumab, was found to highly protective in animal models of infection, including in hamsters, a species that is acutely sensitive to the *C. difficile* toxins [228]. Given the specificity of these antibodies it is not surprising that they have minimal adverse impact on the microbiota [229]. In two large-scale human phase III trials, bezlotoxumab alone was shown to dramatically reduce the rate of recurrence [230], a finding that has been confirmed in later studies [231]. Interestingly, it has recently been shown that bezlotoxumab also blocks extraintestinal organ damage that occurs due to systemic dissemination of the toxins following damage to the intestinal mucosa in a mouse model of CDI [232]. This highlights a potential important application of antibody therapy in ameliorating the worst effects of CDI in severe infections. Bezlotoxumab is a clear success story for monoclonal antibody therapy but this approach is not without limitations, not least challenging production and resulting high cost, estimated to be in excess of $6,000 per patient [233]. One possible avenue to avoid these drawbacks is in the development of therapeutic nanobodies instead. Nanobodies are cheaper to mass produce and can have superior pharmacokinetic properties to traditional monoclonal antibodies [234], leading to significant interest in their potential as therapeutic agents for many pathogenic bacteria, including *C. difficile* (reviewed [235]. Nanobodies that effectively target TcdA or TcdB receptor binding [236] and glucosyltransferase domains [237], the binary toxin [238] and the S-layer [239] have been described but, despite their promise, none of these have progressed to human trials as of yet.

### Novel small molecule antimicrobials

The limited arsenal of *C. difficile* antibiotics, and the additional problems of collateral damage to the microbiome posed by broad-spectrum antimicrobials, has led to a more concentrated search for species-specific antimicrobials. Such narrow spectrum antimicrobials could have several possible advantages over traditional agents, including reduced impact on the microbiome, reduced recurrence rates, superiority over conventional treatment, and improved pharmacokinetic profiles [240]. Despite the intense interest and investment in this space, it has proven challenging to develop antimicrobials of sufficient specificity that are also superior to the current gold standards vancomycin and fidaxomicin. As a result, several promising agents have ceased development after disappointing trial results. One such agent, lacticin 3147, is a two-component lantibiotic produced by *Lactococcus lactis* that targets the cell wall precursor lipid II to inhibit peptidoglycan biosynthesis, as well as forming pores in the cell membrane to achieve cell death [241]. The potent cell killing activities of lacticin 3147 to a range of *C. difficile* isolates was demonstrated in a faecal fermentation model, achieving complete elimination of *C. difficile* in 30 min [242]. However, whilst leaving non-spore-forming anaerobes and total Gram-negative anaerobes intact, this antimicrobial had negative impacts on lactobacilli and bifidobacteria – a likely reason why this treatment has not been taken forward over the last decade [243]. Another promising small molecule antibiotic, cadazolid, has also been abandoned after disappointing phase III results [244]. Cadazolid is a structural hybrid of the oxazolidinone and quinolone classes which had previously demonstrated impressive activity against *C. difficile in vitro* [245] and had performed well in phase II but was inferior to vancomycin in two phase III trials. Surotomycin, a membrane-targeting cyclic lipopeptide with excellent activity against *C. difficile in vitro* [246] was similarly abandoned by Merck following a phase III trial which failed to demonstrate superiority over vancomycin [247]. Despite these recent disappointments, several interesting candidate drugs remain at various stages in the development pipeline. Ibezapolstat, a potent DNA polymerase IIIC inhibitor [248], is currently in phase IIb after a successful initial phase II trial [249]. Ibezapolstat has a favorable pharmacokinetic profile, promoting high concentrations at the site of infection in the colon [249], and appears to induce less harmful changes in microbiota composition and diversity than vancomycin [250].

Ridinilazole, a novel small-molecule antimicrobial with highly specific activities against *C. difficile* [251], is currently the only anti-*C. difficile* antibiotic in phase III (reviewed in [252]). Whilst the mechanism of action of ridinilazole is yet to be fully characterised, phase II trials demonstrated superiority compared to vancomycin. Ridinilazole sustained a 66.7% clinical response rate, compared to 42.4% for vancomycin, and a higher clinical cure rate; whilst showing markedly reduced recurrence rates. Ridinilazole is also poorly absorbed, thus achieving high concentrations in the colon [253]. Recently, *C. difficile* strains from Asia, which were broadly resistant to several antimicrobials, were all shown to be highly susceptible to ridinilazole [252]. Together, these studies suggest great promise for ridinilazole as a novel *C. difficile-*specific antimicrobial agent.

In addition to the novel compounds that are currently in clinical development, there has also been significant interest in repurposing existing licensed drugs. Among these are the antirheumatic agent auranofin [254] and antibiotics such as fusidic acid [255], rifampin [256], and tigecycline [257]. These and further alternative therapeutics are reviewed in depth here [240].

## Conclusions

The increasing disease incidence, coupled with growing reports of community-acquired CDI and the threat of antimicrobial resistance has focussed efforts on characterization of *C. difficile* as an opportunistic pathogen. Over the last decade, work on *C. difficile* has exploded, owing greatly to the ever-expanding array of genetic tools available. Despite such victories, there are still many research gaps to address. Further understanding of virulence factors, resistance mechanisms and host interactions will no doubt aid development of novel therapeutics, and exploring alternative therapeutic avenues may also prove fruitful. It should not be forgotten, however, that the success of *C. difficile* as a pathogen is owed largely to its remarkable genome plasticity – allowing the acquisition of virulence factors and an array of resistance mechanisms. With this in mind, it is clear that the road to combatting this pathogen is far from complete [56,258,259,260,261].
